# RhoMax: Computational Prediction of Rhodopsin Absorption Maxima Using Geometric Deep Learning

**DOI:** 10.1021/acs.jcim.4c00467

**Published:** 2024-06-03

**Authors:** Meitar Sela, Jonathan R. Church, Igor Schapiro, Dina Schneidman-Duhovny

**Affiliations:** † The Rachel and Selim Benin School of Computer Science and Engineering, The Hebrew University of Jerusalem, Jerusalem 9190401, Israel; ‡ Fritz Haber Center for Molecular Dynamics Research, Institute of Chemistry, The Hebrew University of Jerusalem, Jerusalem 9190401, Israel

## Abstract

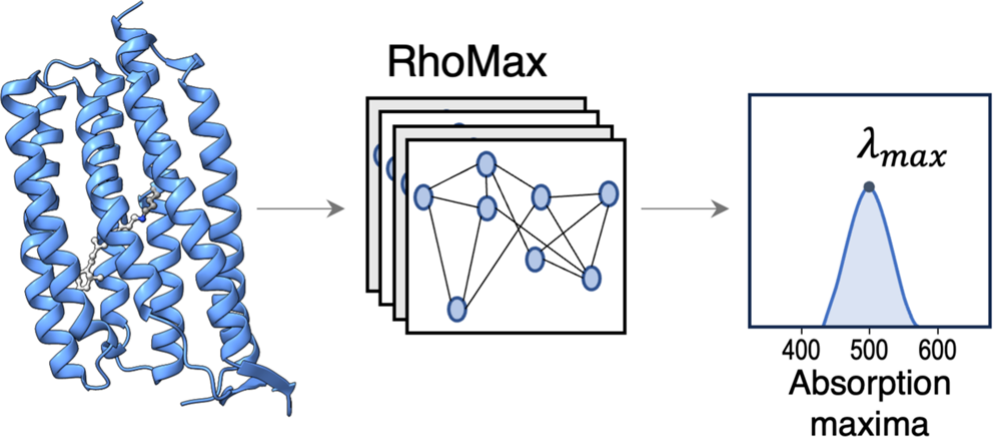

Microbial rhodopsins (MRs) are a diverse and abundant family of photoactive membrane proteins that serve as model systems for biophysical techniques. Optogenetics utilizes genetic engineering to insert specialized proteins into specific neurons or brain regions, allowing for manipulation of their activity through light and enabling the mapping and control of specific brain areas in living organisms. The obstacle of optogenetics lies in the fact that light has a limited ability to penetrate biological tissues, particularly blue light in the visible spectrum. Despite this challenge, most optogenetic systems rely on blue light due to the scarcity of red-shifted opsins. Finding additional red-shifted rhodopsins would represent a major breakthrough in overcoming the challenge of limited light penetration in optogenetics. However, determining the wavelength absorption maxima for rhodopsins based on their protein sequence is a significant hurdle. Current experimental methods are time-consuming, while computational methods lack accuracy. The paper introduces a new computational approach called RhoMax that utilizes structure-based geometric deep learning to predict the absorption wavelength of rhodopsins solely based on their sequences. The method takes advantage of AlphaFold2 for accurate modeling of rhodopsin structures. Once trained on a balanced train set, RhoMax rapidly and precisely predicted the maximum absorption wavelength of more than half of the sequences in our test set with an accuracy of 0.03 eV. By leveraging computational methods for absorption maxima determination, we can drastically reduce the time needed for designing new red-shifted microbial rhodopsins, thereby facilitating advances in the field of optogenetics.

## Introduction

Rhodopsins are transmembrane photoreceptor proteins that are composed of an apoprotein opsin and a retinal chromophore. Retinal forms a covalent bond with a lysine side chain of the opsin, making it a light-responsive protein. These proteins are classified into two types based on their occurrence in organisms: microbial (type I) and animal (type II) rhodopsins.^1^ Microbial rhodopsins have vast biotechnological applications and serve as light-activated photoswitches that precisely control various physiological processes.^2−5^ These proteins are primarily involved in transporting ions across the membrane but also serve as sensors, enzymes, and light-harvesting agents.^6^ The discovery of channelrhodopsins, a type of microbial rhodopsin, was a significant milestone in the development of the optogenetics field.^2,7−9^ Optogenetics is the field of using optical systems and genetic engineering technologies to precisely control and monitor the biological functions of a cell, group of cells, tissues, or organs with high spatial and temporal resolution.^10^ The field has made remarkable achievements in neuroscience, particularly in the precise control of neurons and decoding neural circuits. However, the effectiveness of optogenetic applications is limited by the competing absorption of other chromophores in the tissue such as hemoglobin and myoglobin. To overcome this limitation and extend the utility of optogenetics in tissues, the absorption spectrum of rhodopsins must be shifted toward 700 nm, which is the lower limit of the transparency window in tissues.^11^

**Figure 1:**
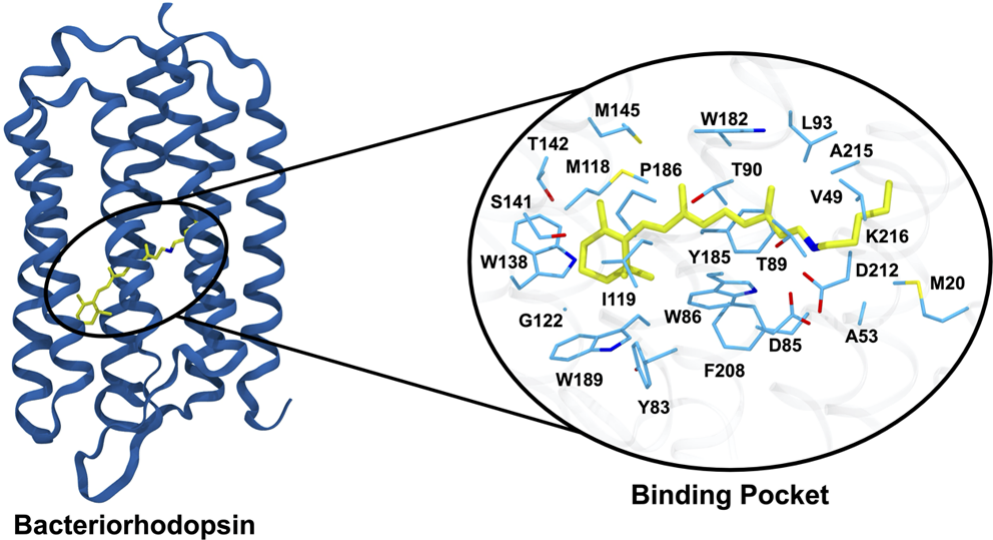
Bacteriorhodopsin structure (PDB 5ZIM) with zoom in on the retinal protonated Schiff base (yellow) and the 24 residues that comprise its binding pocket (light blue).

Typically, opsins absorb in the range between 480 and 600 nm.^12−15^ Red-shifted rhodopsins can be found in nature^16−19^ or designed. However, their experimental characterization and computational studies using quantum chemical methods are demanding and time-consuming. An alternative approach to predict the absorption wavelength rapidly and accurately is highly sought after. A first attempt to utilize machine learning by Inoue et al. predicts the absorption wavelength of a given rhodopsin based on the sequence information and the biochemical features of the amino acid residues.^20^ This approach utilizes the Bayesian LASSO^21^ gradient descent technique to design a regression model based on predefined physicochemical features of amino acids. This approach does not take into account the geometric three-dimensional (3D) structure of the rhodopsin protein (Figure 1), which might also be utilized for accurate prediction of the absorption wavelength.

The low-throughput nature of experimental techniques used to determine the structure of biomolecules, such as X-ray crystallography and cryo-electron microscopy, poses a challenge to the progress of structural knowledge. A further complication arises from the fact that rhodopsins are membrane proteins, which make the experimental structure determination even more challenging. Recently, the field of computational protein structure prediction has undergone a significant breakthrough mainly due to the advancement of deep learning techniques.^22^ AlphaFold2^23^ and RoseTTAFold^24^ have significantly enhanced our ability to predict protein structures with atomic accuracy.^25^ These methods have several standout principles, including utilizing multiple sequence alignment (MSA) to extract residue–residue contacts from evolutionary information,^26−28^ relying on attention-based architectures to capture complex inter-residue relations,^29^ and using an SE(3)-equivariant transformer network^30^ to compute atomic coordinates directly.

The aforementioned breakthrough in structure prediction caused a rise in the utilization of graph neural networks (GNNs) in the field of biochemistry for analyzing properties based on geometrical structures.^31−33^ Graph Neural Networks (GNNs) use message passing between nodes in graphs to discern the interdependent relationships among the graph nodes, making them powerful neural models for analyzing graph structures. A crucial advantage of GNNs is their ability to be customized to display either invariance or equivariance to SE(3) transformations, thereby making them ideal for learning molecular properties. Over time, there have been improvements and variations in GNNs, including graph convolutional networks (GCNs), graph attention networks (GATs), and graph recurrent networks (GRNs), which have demonstrated remarkable performance in different deep learning tasks.^34−38^ Geometric deep learning has far-reaching implications in various biotechnological domains,^39,40^ such as protein docking,^41−44^ molecule design,^45−48^ and property prediction.^33,49−51^ Its advancements have revolutionized the field of protein structure determination, bringing about a paradigm shift and creating new prospects for progress in biotechnology and pharmaceuticals.

Here, we present RhoMax, a novel machine learning approach that employs geometric deep learning techniques to predict the absorption wavelength of rhodopsins. It is a GNN model that relies on the geometric 3D structure of rhodopsins as an input, which is calculated using AlphaFold2,^23^ and predicts the corresponding maximum absorption wavelength in nanometers. The model is designed to be invariant under Euclidean-rigid transformations and uses attention layers to better capture the relationships between different atoms. Our model is trained and tested using a data set by Inoue et al., comprising 884 sequences,^20,52^ and can predict the wavelength of a given structure within seconds. RhoMax predicts the absorption wavelength with an average accuracy of 6.8 nm on the test set sequences.

## Materials and Methods

### Method Summary

**Figure 2:**
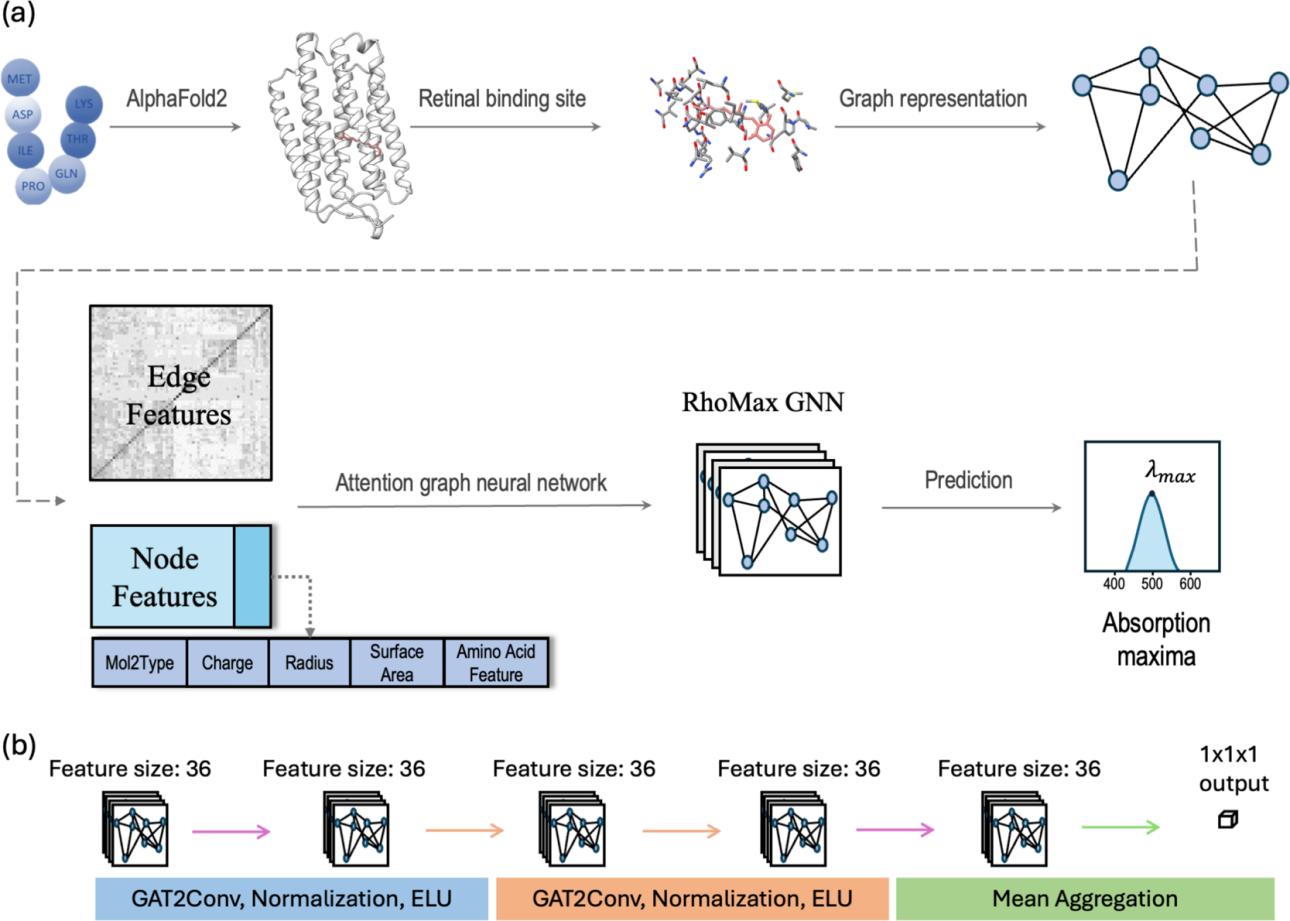
RhoMax model representation (a) and network architecture (b).

The input to RhoMax is the rhodopsin sequence, and the output is the corresponding maximum absorption wavelength (Figure 2A). AlphaFold2^23^ is employed to create a three-dimensional structure, and multiple sequence alignment is applied to extract the 24 residues located around the retinal chromophore as proposed in the previous work by Inoue et al.^20^ These amino acids constitute the retinal binding pocket and are used to train a deep neural network, RhoMax, based on a graph attention model. Here, each atom that belongs to the amino acid of the binding pocket is represented by a node, while atomic bonds are represented as edges between atoms if they fall within a threshold of 2 Å. Every node is assigned a feature vector that characterizes its spatio-chemical properties, and the edge features are chosen according to the geometric distance between the atoms. We have used the data set from Inoue et al. composed of 884 rhodopsin sequences,^15,20^ which were utilized to train and validate the network. The loss function of the model quantifies the difference in nanometers between the predicted absorption maximum and the experimental counterpart.

### Data Set

**Figure 3:**
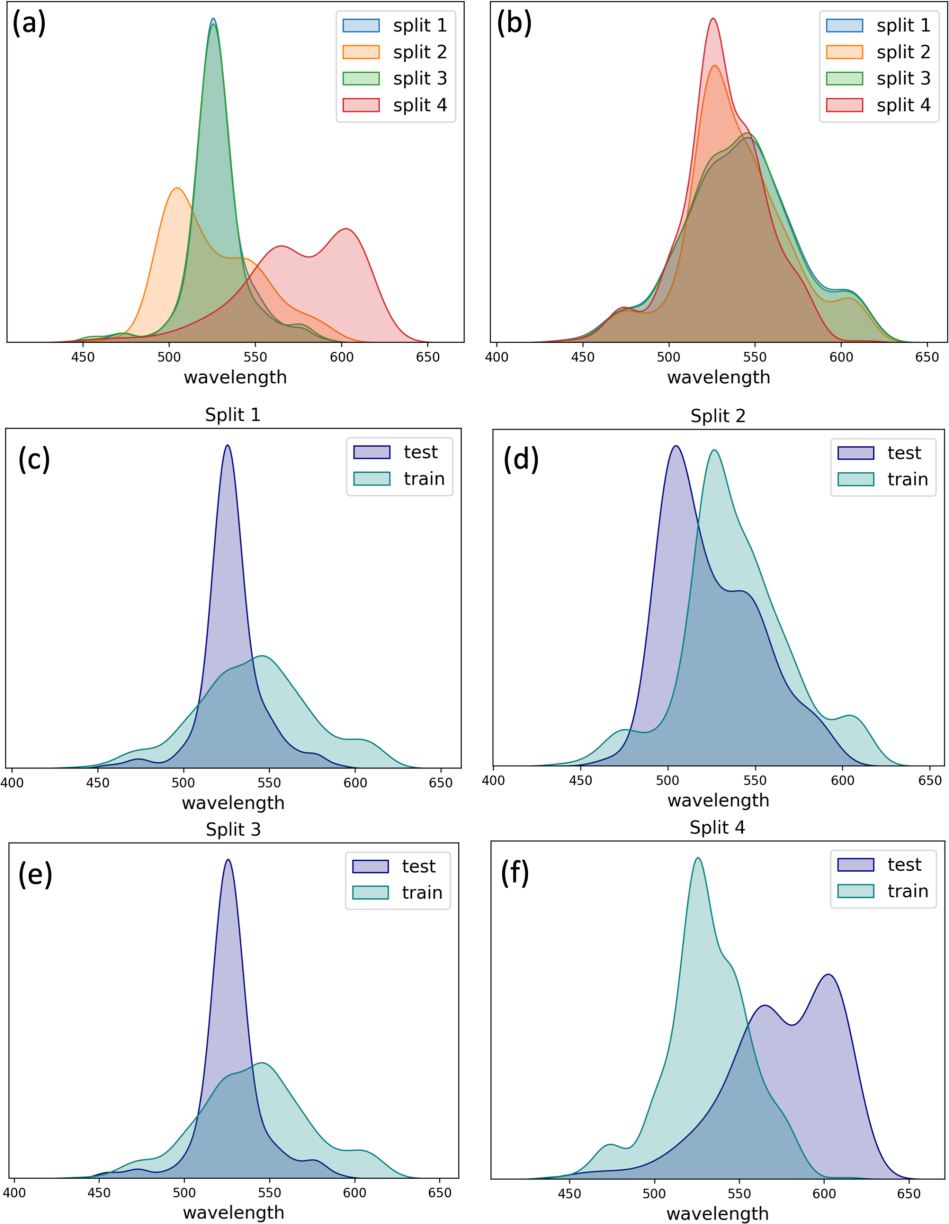
Distribution of absorption maxima in different data sets. (A) distribution in the test sets from splits 1–4. (B) distribution in the train sets from splits 1–4. Panels (C–F) illustrate the distribution comparison in the train/test splits 1–4, respectively.

**Figure 4:**
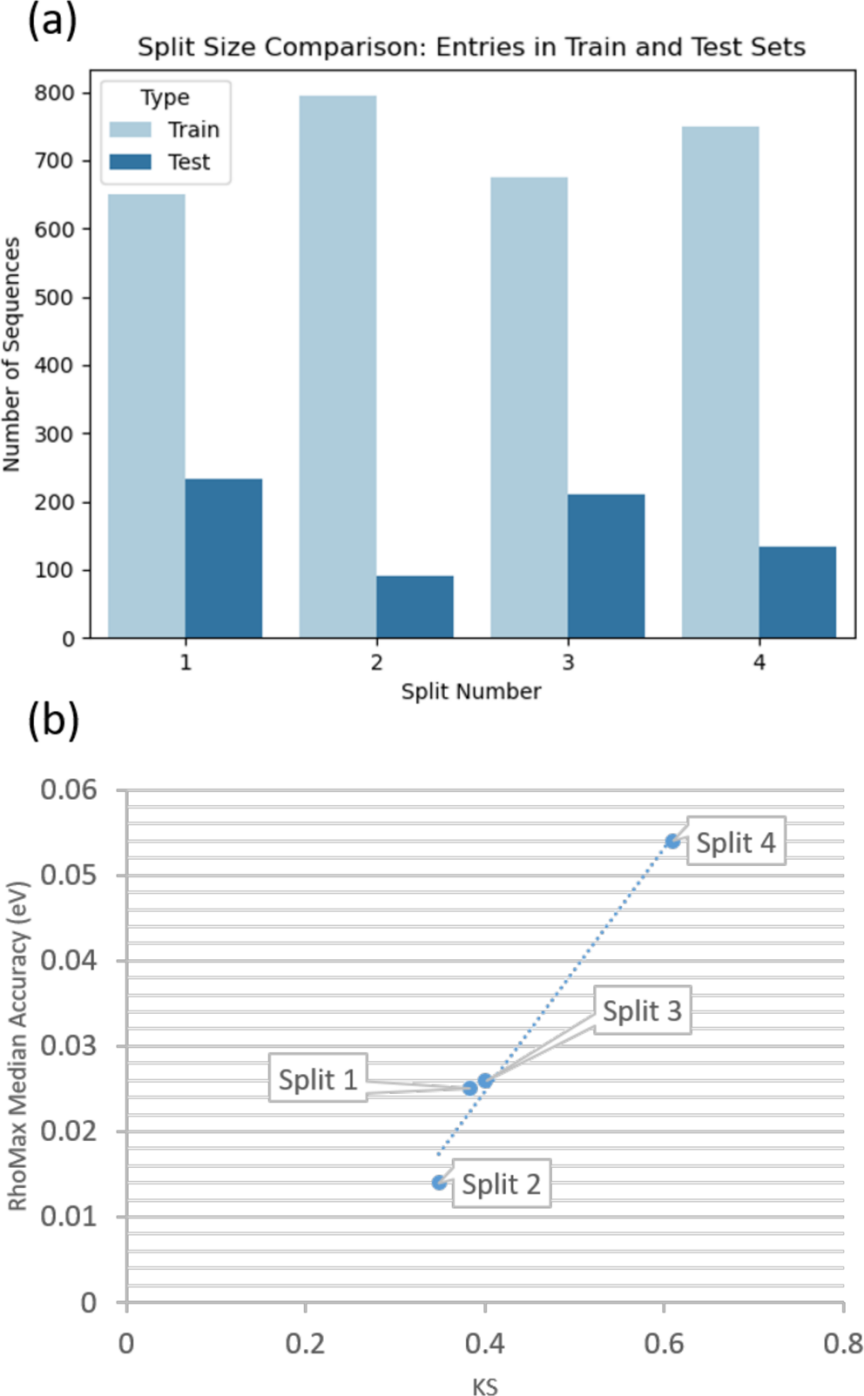
(a) Number of sequences in the train and test sets for each of the four data splits. (b) RhoMax median accuracy vs train-test absorption maxima similarity as measured by the Kolmogorov–Smirnov (KS) test.

We use a data set that contains 75 wild-type (WT) microbial rhodopsin sequences compiled by Inoue et al., with several mutations for each WT leading to a total of 884 sequences.^20,52^ Every record in this data set contains the WT or mutant sequence, the maximum absorption wavelength, and the experimental method used to determine it. The distribution of the absorption maxima in this data set is not uniform, with most of the rhodopsins in the range of 500–570 nm (Figure 3A). In response to this significant imbalance, we compute four distinct splits of training and testing sets to address this variability (Figure 3C–F). Because most mutant sequences have only one or two mutations, we decided that they would be assigned to the same set (train or test) as their WT to avoid data leakage between the train and test sets. Each split consists of 65 and 10 WT sequences in the train and test sets that were divided randomly, respectively. Since the number of mutant sequences for each WT is different, the size of train and test sets is not identical for each data split (Figure 4). Moreover, this data split protocol does not guarantee that the absorption maxima values have similar distributions in the train and test sequences (Figure 3C–F).

### Energy Units

The spectroscopic measurements of the absorption spectra of rhodopsins are reported in wavelength for historical reasons. The absorption maxima are reported units of nanometers (nm). However, the wavelength is inversely proportional to energy by definition: , where *E* is the energy of light, *h* is the Planck constant, ν is the frequency, *c* is the speed of light, and λ is the wavelength. As electronvolts (eV) are linear in energy, they are better units for prediction. We have provided the results in both units to facilitate comparison with previous reports.

### Protein Structure Generation and Representation

We generated five structures for each sequence using AlphaFold2^23^ through ColabFold^25^ with default parameters and without templates (alphafold-ptm v1.0). This protocol results in structural models with an estimated median RMSD of 1.08 Å as measured on the sequences with available crystal structures in the PDB (Table S1). We have selected the highest-ranked structure for further processing and narrowed the model’s focus to the retinal binding pocket (Figure 1). In total, 24 amino acids constitute the binding pocket, which has been previously determined based on close proximity to the retinal by Inoue et al.^20^ Their importance was confirmed in previous studies.^20^ We use multiple sequence alignment established through ClustalW^53^ to identify the positions of the binding pocket residues. Because AlphaFold2 is limited to protein amino acids, we train RhoMax on structures without the retinal. Our assumption is that the chromophore geometry is dictated by the protein structure and is anchored to the same conserved lysine position. Therefore, the retinal is implicitly considered in this setting. In addition, we train a second model on structures with chromophores that were generated by copying the retinal from the closest homologues in the PDB via structural alignment of rhodopsin.

In our deep learning model, we represent the 3D geometry of the input retinal binding pocket as a graph (Figure 2A). The nodes of the graph correspond to the atoms, and the edges connect nearby atoms. We connect atoms that are less than 2 Å apart to include all the chemical bonds, resulting in a sparse graph. We set the edge feature of a connection between atoms *x*_*i*_ and *x*_*j*_ to be , where *d*(*x*_*i*_, *x*_*j*_) is the Euclidean geometric distance function in angstroms between two nodes *x*_*i*_, *x*_*j*_. Features that remain constant across the training set are removed, while each of the remaining features is normalized to a normal distribution.

Each atom in the graph is represented by a features vector of size 36. The first 18 values were adopted from Inoue et al., and they correspond to the chemical properties of the amino acid that the atom is part of, including organic and inorganic value, hydropathy, polarity, isoelectric point, molecular weight, volume, etc. The next 15 values of this vector encode the atom type based on the tripos 5.2 force field.^54^ Such one-hot encoding for categorical data involves representing each atom as a binary vector, where each position corresponds to a specific atom type, and only one atom type is set to 1, while others are set to 0. The last three values are partial atomic charges from the CHARMM22 force field,^55^ solvent accessible area per atom, and atomic radius.^56^

### Network Architecture

The network architecture consists of four graph convolutional layers (Figure 2B). The first and the last layers are Graph Convolutional layers, as implemented in pytorch.^34^ The second and the third layers are graph attention convolutional layers.^35^ All the layers do not include self-loops and use only one head. After each layer, we apply batch normalization^57^ and Exponential Linear Unit (ELU).^58^ At the end, we use mean aggregation to output a single scalar output (Figure 2B). In graph neural networks, each node within a layer combines information from its neighbors using an aggregation function and subsequently forwards it to the next layer, updating its features accordingly. Consequently, nodes that are not connected by edges (distance >2 Å) tunnel their information through neighboring nodes, thereby ensuring that their relationships are learned by the network. Thus, through the utilization of graph neural networks, we inherently account for noncovalent interatomic interactions. The total number of trained parameters in the network is 6,401. Larger data sets will enable adding additional layers to train deeper models.

### Loss Function

For a given prediction in nanometers *y* and a true value in nanometers *y*_0_, we define the loss function as *L*(*y*, *y*_0_) = |*y* – *y*_0_|, and we minimize it during training.

### Training Details

During the training process, we utilized the Adam optimizer with a weight decay of 0.00001 for L2 regularization, as well as an α parameter of 0.0001 for L1 regularization. Additionally, we set the learning rate to 0.0001 and trained the model for 1000 epochs.

### Comparison to Previous Methods

We compare our results to the previous method of Inoue et al.^20^ that relies on the BLASSO (Bayesian LASSO) regression approach. We used their R code to train and test their model on the same data set splits that we used in the present work (Figures 3 and 4). While the original model was trained in nanometers, we have updated the code to support eV training.

## Results

### RhoMax Performance

**Figure 5:**
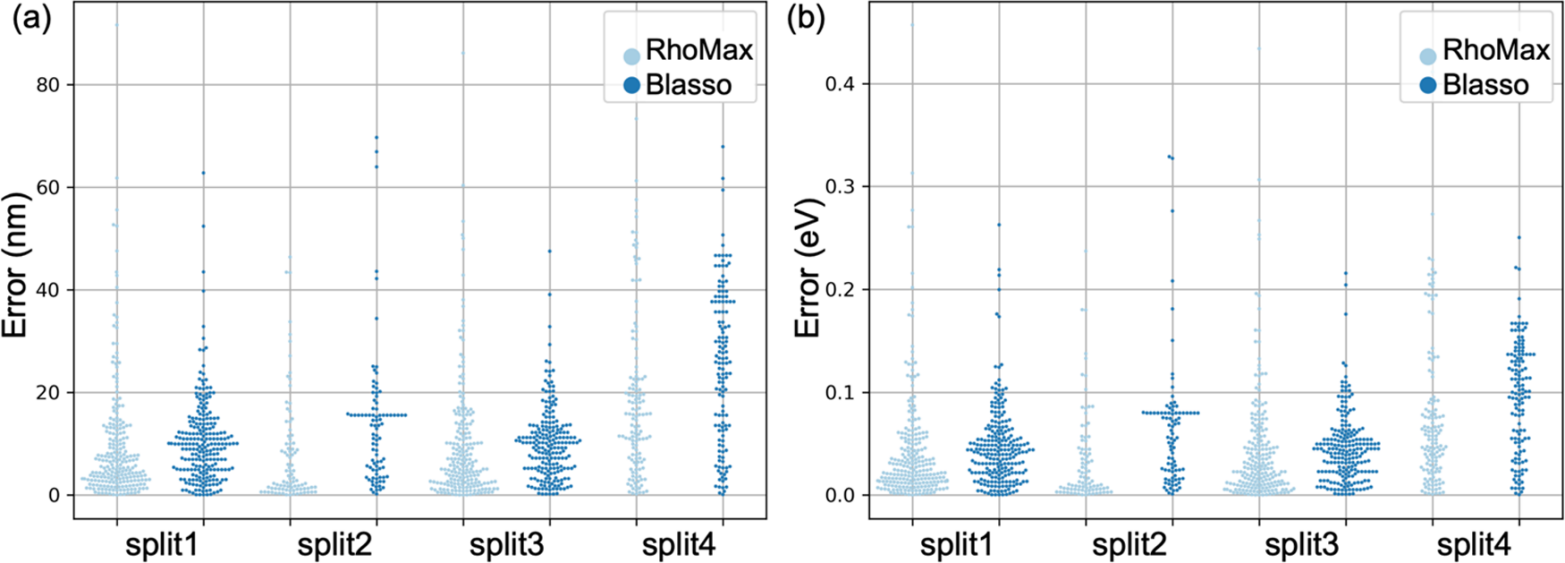
Performance of RhoMax (light blue) and Blasso (dark blue) on the test set for each of the four splits. The error is measured by a difference between the predicted and experimentally measured absorption maxima in nm (a) or eV (b).

Our new model, trained on structures without explicitly including retinal, predicts absorption maxima with a median accuracy of about 0.03 eV, which corresponds to 6.83 nm (Table 1). There is variability in accuracy across the splits due to the data set’s limited size, causing differing spectra value distributions in the training and test sets for each split (Figure 3). The lowest accuracy is found for split number four, where the test set contains mostly red-shifted rhodopsins (Figure 3F). However, the model was trained on blue-shifted rhodopsins and therefore predicted less accurate results for sequences with absorption maxima at longer wavelengths. We find that the accuracy of the model is correlated with the similarity between the absorption maxima distributions in the train and test sets (Figure 4B). The distributions were compared using the Kolmogorov–Smirnov test (as implemented in the Scipy library). The model that was trained on structures with the retinal had slightly lower accuracy (Table 1). Consequently, we continue our analysis only on the model trained on structures without the retinal (Figure 5).

**Table 1:** Performance of RhoMax and BLASSO on the Test Set for Each of the Four Splits for eV and nm

	median			mean and standard deviation		
eV	RhoMax	RhoMax + retinal	BLASSO	RhoMax	RhoMax + retinal	BLASSO
split 1	0.025	0.027	0.047	0.045 ± 0.06	0.046 ± 0.06	0.047 ± 0.04
split 2	0.014	0.036	0.058	0.034 ± 0.04	0.045 ± 0.04	0.064 ± 0.06
split 3	0.026	0.026	0.043	0.045 ± 0.06	0.045 ± 0.05	0.045 ± 0.03
split 4	0.054	0.090	0.100	0.074 ± 0.07	0.090 ± 0.06	0.095 ± 0.05
mean	0.030	0.045	0.062	0.050 ± 0.06	0.057 ± 0.05	0.063 ± 0.05

nm	RhoMax	RhoMax + retinal	BLASSO	RhoMax	RhoMax + retinal	BLASSO
split 1	5.20	6.14	9.94	8.85 ± 10.5	10.29 ± 12.2	10.58 ± 8.4
split 2	3.40	7.95	13.55	7.28 ± 10.2	10.24 ± 9.3	13.89 ± 13.0
split 3	5.32	5.85	10.12	8.98 ± 10.9	9.99 ± 11.6	10.10 ± 7.0
split 4	13.40	23.74	25.70	16.68 ± 14.6	23.33 ± 13.7	25.02 ± 15.1
mean	6.83	10.92	14.83	10.45 ± 11.6	13.46 ± 11.7	14.90 ± 10.9

### Comparison to Previous Methods

We have compared RhoMax’s performance to the previously published approach that relied exclusively on sequential data and used Bayesian LASSO^21^ for model training.^20^ RhoMax doubles the accuracy compared to the Bayesian LASSO (Figure 5 and Table 1). This improvement can be attributed to our model’s ability to incorporate geometric information at an atomic level represented as a graph, as well as additional features (mol2 type, radius, surface area, and atomic charge) and attention layers that aid in capturing interatomic relationships.

### Ablation Studies

**Figure 6:**
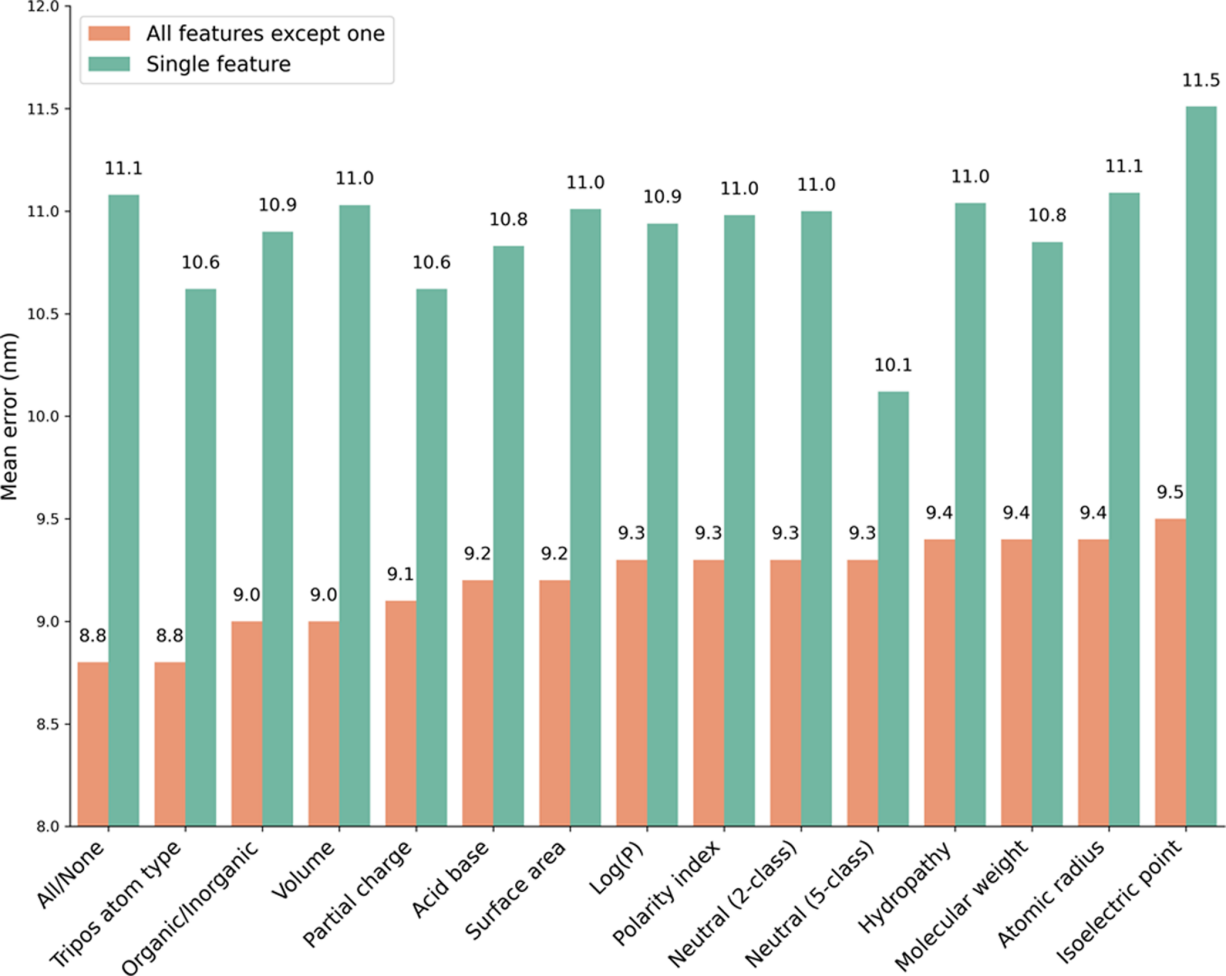
Mean error (nm) for RhoMax ablation tests on split 1. The orange bars correspond to the prediction error when all but one feature is included, while the green bars correspond to the results when only a single feature is considered or no features at all for the leftmost bar.

To evaluate the physicochemical features that contribute to prediction accuracy, we performed several ablation experiments on the train-test split number 1. In these experiments, different components or features of a model are removed or disabled to understand their impact on the model’s performance. The first type of ablation tested the effect of removing a single feature on the accuracy of the RhoMax model, while the second type trained a model by removing all but one single feature (Figure 6 and Table 2). We could not perform ablation experiments of the structural information because it is encoded in the graph representation. Therefore, the contribution of the geometry to the overall accuracy can be evaluated separately.

**Table 2:** Mean Nanometer Loss Results Obtained from RhoMax Ablations on Split 1

feature	all features but one	only one feature
all/none	8.8	11.1
acid base property	9.2	10.8
partial atom charge (CHARMM22)	9.1	10.6
neutral (5-class)^a^	9.3	10.1
hydropathy	9.4	11.0
isoelectric point	9.5	11.5
log(*P*)	9.3	10.9
tripos 5.2 atom type	8.8	10.6
molecular weight	9.4	10.9
organic/inorganic value	9.0	10.9
polarity index	9.3	11.0
neutral (2-class)	9.3	11.0
atomic radius	9.4	11.1
surface area	9.2	11.0
atomic volume	9.0	11.0

a Neutral amino acid side chains were classified in two different ways. In the 2-class classification, the neutral side chains are separated into polar and nonpolar groups. In the 5-class classification, the following categories are available: aliphatic, aromatic, neutral, cysteine, and proline/glycine.

Overall, removing a single feature has a nonsignificant effect. The average predicted absorption maximum has not deteriorated by more than 1 nm for any feature ablation (Figure 6, orange bars). The second type of ablation revealed similar changes with respect to the reference value of having no features at all. We find that the most significant effect was noted for the feature that classifies each amino acid into one of five types of neutral residues. This feature distinguishes between aliphatic, aromatic, and neutral side chains while having separate categories for cysteine as well as proline and glycine. Including only this single feature changed the results by nearly 1 nm on average. All the other features have a significantly smaller effect. Overall, we do not find features with significant contributions to the prediction accuracy, indicating that there is a synergetic effect between the features. A reasonable prediction could be obtained even with single features or without features at all, indicating that the geometric configuration plays an important role. Without the features, the prediction is made solely based on the graph structure that represents the geometry of the binding pocket.

To evaluate the impact of the attention mechanism within the network, we conducted experiments where we maintained the same network architecture but substituted the graph attention layers with regular graph convolutional layers (Table 3). It is evident that utilizing attention layers enhances RhoMax performance and yields more accurate outcomes.

**Table 3:** Performance of RhoMax Without the Attention Layer for Each of the Four Splits for eV and nm

	median (eV)	mean and standard deviation (eV)	median (nm)	mean and standard deviation (nm)
split 1	0.025	0.045 ± 0.06	5.55	10.14 ± 12.7
split 2	0.024	0.046 ± 0.05	5.21	10.45 ± 11.4
split 3	0.024	0.048 ± 0.06	5.47	10.58 ± 13.4
split 4	0.072	0.088 ± 0.07	20.33	22.63 ± 16.4
mean	0.036	0.056 ± 0.06	9.14	13.45 ± 13.5

## Discussion

Optogenetics is limited by the fact that blue light has a low ability to penetrate biological tissues, and discovering additional red-shifted rhodopsins could be a significant advancement in this field. The ability of a computational approach to predict absorption wavelength quickly and accurately could be immensely beneficial in reducing the number of experiments necessary for designing red-shifted rhodopsins. In this study, we introduced a new computational approach called RhoMax, which utilizes geometric deep learning techniques to predict the maximum absorption wavelength of microbial rhodopsins (MRs) based on sequence information. Our results demonstrate that RhoMax is capable of accurately predicting the maximum absorption wavelength of over half of the sequences in our test set with an accuracy of less than five nanometers. This represents a considerable improvement over previous approaches that relied only on sequences and utilized BLASSO for model training (Figure 5).

However, our study also has limitations. First, the size of the data set used to train the model was limited to 884 sequences by Inoue et al.^20,52^ due to a lack of available experimental absorption maxima. This is exemplified in the differing spectra distributions between the training and test sets and the variability in the accuracy of the predicted wavelengths depending on how the data is split (Figure 3). Training RhoMax on a larger data set may lead to even greater accuracy or at least produce more consistent theoretical results. In our model, which was trained on structures with chromophores, the accuracy was lower (Table 1), indicating that incorporating the retinal in the predicted structures is not a trivial process because conformational relaxation is required. For example, some crystal structures of microbial rhodopsins show the β-ionone ring of the retinal rotated from 6-s-*trans* to 6-s-*cis.*^59^ Given that we positioned the retinal based on the structure of the closest homologue in the PDB, inaccuracies in the placements are expected and they adversely affect the model’s performance. We anticipate that additional structures and the recent introduction of cofolding approaches^60^ will enable more accurate retinal placement in the future.

We believe that the use of RhoMax can assist in the determination of absorption maxima during the development of new red-shifted microbial rhodopsins, which is a critical component in the advancement of optogenetics. One possible application could be screening of metagenomics of rhodopsin sequences, which has recently led to the discovery of new families.^14,16^ Alternatively, new rhodopsins can be designed using novel sequence- or structure-based deep learning models. Sequence-based design methods usually rely on unsupervised protein language models. Conditional models, such as ProGen,^61^ can be used to generate new sequences that belong to the rhodopsin family. Structure-based design models, such as ProteinMPNN,^62^ can be utilized to computationally design red-shifted rhodopsins^63−65^ using known structures as a scaffold. The designed sequences are usually structurally validated using structure prediction methods, such as AlphaFold, followed by experimental methods that verify selected sequences^66^ with the highest structural confidence and desired range of predicted absorption maxima.

## Data Availability

The data sets, source code, and a Colab notebook that runs RhoMax are available from the GitHub repository: https://github.com/dina-lab3D/OpsiGen/

## Supporting Information Available

Validating AlphaFold2 on rhodopsin sequences with known structures. Ca RMSD between the best scoring model and PDB structure (PDF)
