# Supplementary material for: RhoMax: Computational Prediction of Rhodopsin Absorption Maxima Using Geometric Deep Learning
Source: J Chem Inf Model. 2024 Jun 3;64(12):4630–9. doi: 10.1021/acs.jcim.4c00467 (PMC11200256; doi:10.1021/acs.jcim.4c00467)
Supplement: Supplementary file 1 — ci4c00467_si_001.pdf [file ci4c00467_si_001.pdf]

Supporting Information

RhoMax: Computational Prediction of Rhodopsin Absorption Maxima using Geometric Deep Learning

Meitar Sela,[1] Jonathan R. Church,[2] Igor Schapiro,[2*] Dina Schneidman-Duhovny[1*]

[1]The Rachel and Selim Benin School of Computer Science and Engineering, The Hebrew University of Jerusalem, Jerusalem 9190401, Israel

[2]Fritz Haber Center for Molecular Dynamics Research, Institute of Chemistry, The Hebrew University of Jerusalem, Jerusalem 9190401, Israel

[*]Correspondence: dina.schneidman@mail.huji.ac.il (D.S.), igor.schapiro@mail.huji.ac.il (I.S.)

Table S1. Validating AlphaFold2 on rhodopsin sequences with known structures. Ca RMSD between the best scoring model and PDB structure.

**WT sequence**	**PDB code**	**RMSD (Å)**
AcetR1	5AWZ	0.73
AR3	6GUX	3.95
CrChR2	6EID	1.83
ESR	4HYJ	1.37
ASR	1XIO	1.26
GPR	7B03	1.69
GR	6NWD	1.51
GtACR1	6EDQ	2.32
HmBRI	4PXK	0.65
HsHR	2JAF	0.89
AR1	1UAZ	0.67
HwBR	5ITC	0.79
KR2	6RF0	2.64
BR	1M0K	0.78
NmClR	6AB9	1.08
XR	3DDL	1.02
AR2	3WQJ	0.54
**Mean**		**1.40**
**Median**		**1.08**
